# A bioenergetic shift is required for spermatogonial differentiation

**DOI:** 10.1038/s41421-020-0183-x

**Published:** 2020-08-18

**Authors:** Wei Chen, Zhaoran Zhang, Chingwen Chang, Zhichang Yang, Pengxiang Wang, Haihui Fu, Xiao Wei, Eric Chen, Suxu Tan, Wen Huang, Liangliang Sun, Ting Ni, Yi Yang, Yuan Wang

**Affiliations:** 1 Shanghai Key Laboratory of Regulatory Biology, Institute of Biomedical Sciences and School of Life Sciences, East China Normal University, Shanghai, 200241 China; 2 Department of Animal Sciences, College of Agriculture and Natural Resources, Michigan State University, East Lansing, MI 48824 USA; 3 Department of Chemistry, College of Natural Science, Michigan State University, East Lansing, MI 48824 USA; 4 State Key Laboratory of Genetic Engineering & MOE Key Laboratory of Contemporary Anthropology, Collaborative Innovation Center of Genetics and Development, School of Life Sciences, Fudan University, Shanghai, 200438 China; 5 Synthetic Biology and Biotechnology Laboratory, State Key Laboratory of Bioreactor Engineering, School of Pharmacy, East China University of Science and Technology, Shanghai, 200237 China

**Keywords:** Stem cells, Cell biology

## Abstract

A bioenergetic balance between glycolysis and mitochondrial respiration is particularly important for stem cell fate specification. It however remains to be determined whether undifferentiated spermatogonia switch their preference for bioenergy production during differentiation. In this study, we found that ATP generation in spermatogonia was gradually increased upon retinoic acid (RA)-induced differentiation. To accommodate this elevated energy demand, RA signaling concomitantly switched ATP production in spermatogonia from glycolysis to mitochondrial respiration, accompanied by increased levels of reactive oxygen species. Disrupting mitochondrial respiration significantly blocked spermatogonial differentiation. Inhibition of glucose conversion to glucose-6-phosphate or pentose phosphate pathway also repressed the formation of c-Kit^+^ differentiating germ cells, suggesting that metabolites produced from glycolysis are required for spermatogonial differentiation. We further demonstrated that the expression levels of several metabolic regulators and enzymes were significantly altered upon RA-induced differentiation, with both RNA-seq and quantitative proteomic analyses. Taken together, our data unveil a critically regulated bioenergetic balance between glycolysis and mitochondrial respiration that is required for spermatogonial proliferation and differentiation.

## Introduction

Spermatogonial stem cells (SSCs) maintain a pool of undifferentiated progenitor spermatogonia during spermatogenesis, the postnatal germ cell development^1^. During this process, undifferentiated spermatogonia proliferate transiently and respond to developmental cues, such as retinoic acid (RA) to become differentiating spermatogonia, which further develop into spermatocytes and haploid spermatids through meiosis^1–5^. It is therefore critical to understand how spermatogonial proliferation and differentiation are balanced to sustain proper spermatogenesis. SSCs are rare and only occupy ~0.03% of germ cell populations in adult mice^1–5^. In contrast, in vitro cultured spermatogonia can proliferate long-term, and thus represents a feasible platform to investigate both genetic and environmental influence on postnatal germ cell development^6,7^. CD90 and CD9 surface antigens are often used to enrich undifferentiated spermatogonia and SSCs (refs. ^8,9^) that express marker genes, such as ID4, GFRα1, and PLZF at high levels^3–5,10–14^. The c-Kit expression marks the appearance of differentiating spermatogonia and early-stage spermatocytes^15^. FGF2 and GDNF are required to maintain spermatogonial proliferation^4,6,16–18^, whereas upon treatment with RA (refs. ^19,20^), CD90^+^/CD9^+^/c-Kit^−^ spermatogonia start to differentiate into c-Kit^+^ cells, in which STRA8 and SYCP3 are upregulated^11,12,14,19–23^. Undifferentiated spermatogonia with an SSC capacity can be further evaluated for their potential to undergo meiosis and regenerate complete spermatogenesis in vivo by testicular transplantation into *Kit*^*w/w-v*^ mice, a commonly used recipient mouse model with defective endogenous germ cell development^24–26^.

All mammalian cells produce ATP with various proportional contributions from glycolysis and mitochondrial oxidative phosphorylation (OXPHOS). These cellular processes not only generate energy, but also provide essential metabolic intermediates that are needed for cell division, lineage development, and epigenetic modifications^27,28^. A tightly regulated balance between glycolysis and OXPHOS is particularly important for stem cell self-renewal and differentiation. It has been shown that the activation of glycolysis and inhibition of OXPHOS increase the reprograming efficiency of somatic cells toward induced pluripotent stem cells^28–30^. Specifically for germ cells, accumulating evidence suggests that an intricate network of transcriptional factors, metabolic drivers, and signaling molecules acts together to maintain a biogenetic balance between glycolysis and OXPHOS (refs. ^27,31,32^). For example, single-cell RNA-seq analyses have revealed differential expressions of metabolic drivers during different stages of spermatogenesis^33,34^. In addition, forkhead box (FOX)O1 regulates the expression of MYC/MYCN transcription factors that in turn modulate cell cycle and glycolysis to impact SSC self-renewal^35^.

Most studies suggest that adult stem cells reside at a niche with low oxygen tension, and thus rely on glycolysis for energy production to avoid DNA damage caused by reactive oxygen species (ROS) from OXPHOS (refs. ^27,31^). However, SSCs and undifferentiated spermatogonia are located at the base of seminiferous tubules with layers of spermatocytes and spermatids sequentially migrating from basal toward luminal regions^1–5^. As blood vessels run between seminiferous tubules, oxygen reaches to the lumen only by diffusion^36,37^. Compared to meiotic and post-meiotic germ cells, spermatogonia appear to have relatively easier access to oxygen^36,37^. Indeed, it was reported that ROS was required for mouse SSC self-renewal by activating P38/MAPK and JNK pathways^38^. However, recent studies also showed that hypoxia culture conditions, and increased glycolysis favored SSC establishment and long-term maintenance^35,39^. It remains undetermined whether the balance between glycolysis and OXPHOS changes during spermatogonial differentiation, and whether a high level of OXPHOS is required for this process.

We hereby conducted experiments to unveil metabolic changes and regulatory mechanisms in spermatogonial differentiation. Using an in vitro differentiation platform, we found that undifferentiated spermatogonia adapted a distinct bioenergetic preference from their differentiating populations. In addition, we identified several novel metabolic regulators that were differentially expressed in undifferentiated and differentiating spermatogonia. Disturbed bioenergetic balance led to the disrupted spermatogonial proliferation and differentiation, thereby supporting an essential role of metabolic regulation in spermatogonial fate specification.

## Results

### Establishing an in vitro spermatogonial differentiation platform

**Fig. 1:**
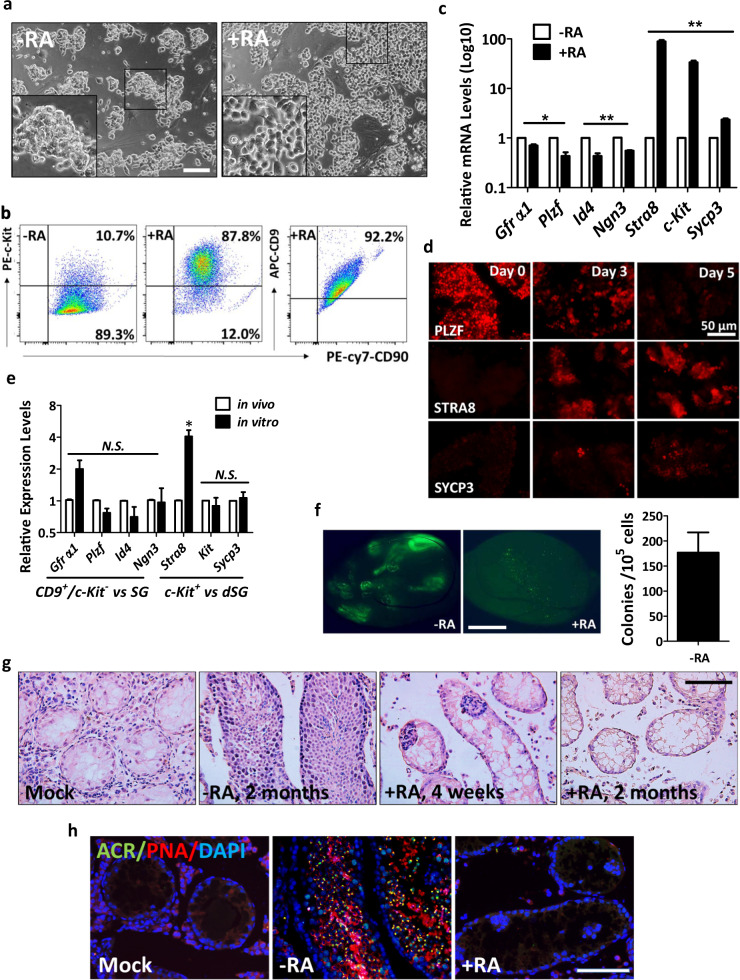
Establishing spermatogonial culture and in vitro differentiation platform. **a** A typical view of undifferentiated (−RA) and differentiating (+RA) spermatogonial clones. Scale bar, 100 μm. Insets are blow-up images with higher magnificent powers. **b** Spermatogonia in culture and upon RA-induced differentiation were analyzed by flow cytometry. **c**, **d** Marker gene expressions in undifferentiated and differentiating spermatogonia were examined by real-time RT-PCR (**c**) and IF (**d**) assays. **e** Marker gene expressions of in vitro cultured spermatogonia (SG) and differentiating population (dSG) were compared with in vivo developed CD9^+^/c-Kit^−^ and c-Kit^+^ cells, respectively, by real-time PCR assays. **c**, **e** Data are presented as mean ± SEM. *P < 0.05; **P < 0.01; N.S., no statistical significance; *n* = 3. **f** Seminiferous tubule transplantation of in vitro cultured GFP^+^ spermatogonia with or without RA treatment into *Kit*^*w/w-v*^ testes. Green fluorescence in testes was detected at two-month post transplantation. A bar graph on the right shows the number of colonies/10−e5 transplanted cells averaged from three recipients. Scale bar, 1 mm. **g***Kit*^*w/w-v*^ testes transplanted with undifferentiated spermatogonia and those with RA treatment. Histology was performed at four-week or two-month post transplantation. **h***Kit*^*w/w-v*^ testes transplanted with undifferentiated spermatogonia and those treated with RA. IHF assays with an ACR antibody, PNA, and DAPI staining were performed at two-month post transplantation. **g**, **h** Scale bar, 100 µm.

To understand metabolic changes during postnatal germ cell development, we first established an in vitro spermatogonial culture and differentiation platform. Undifferentiated spermatogonia exhibited typical clustered grape-like clones^6,7^ (Fig. 1a) and ~90% of them expressed the surface antigens CD90 and CD9, assessed by flow cytometry analyses (Fig. 1b). Upon RA-induced differentiation, the cells in spermatogonial colonies became loosely connected (Fig. 1a) and started to differentiate into c-Kit^+^ cells (Fig. 1b). In addition, undifferentiated spermatogonia highly expressed SSC markers, such as ID4, GFRα1, and PLZF that were downregulated in response to RA treatment (Fig. 1c, d). By contrast, c-Kit, STRA8, and SYCP3 were significantly increased at both RNA and protein levels in RA-induced c-Kit^+^ differentiating cells (Fig. 1b–d). No statistically significant differences were found in the expression of marker genes (*Gfrα1, Plzf, Id4*, and *Ngn3*) between in vitro maintained spermatogonia and in vivo developed CD9^+^/c-Kit^−^ cells (Fig. 1e). Similarly, *c-Kit* and *Sycp3* displayed comparable levels between in vitro RA-treated population and c-Kit^+^ cells collected from testes (Fig. 1e). However, we observed increased *Gfrα1* (albeit not statistically significant) and *Stra8* expression levels from in vitro cultured cells compared to their in vivo counterparts (Fig. 1e). One potential explanation may relate to the high heterogeneity of in vivo isolated populations. For example, isolated c-Kit^+^ cells include differentiating spermatogonia at different developmental stages that express *Stra8* at various levels. CD9^+^/c-Kit^−^ cells contain undifferentiated spermatogonia at different developmental stages and may also be contaminated with a few somatic cells (e.g., mesenchymal cells) that express CD9 (ref. ^40^). In vitro culture favors the growth of undifferentiated spermatogonia and synchronizes them to respond to RA treatment in a more homogenous manner. Another possibility of elevated *Stra8* expression may be due to the high RA induction dose in vitro (Supplementary Fig. S1a). Nevertheless, undifferentiated spermatogonia that were maintained for more than four months in vitro robustly reconstituted spermatogenesis after seminiferous tubule transplantation, and formed ACR (Acrosin)+ and PNA+ haploid spermatids in testes of *Kit*^*w/w-v*^ mice (Fig. 1f–h), a model with defective endogenous germ cell development^24–26^. By contrast, only residue derivatives from differentiating spermatogonia in the RA-treated group were observed at four weeks, but no spermatids were detected at two-month post transplantation (Fig. 1f–h), suggesting efficient differentiation induced by RA. Taken together, we successfully established an in vitro differentiation platform of spermatogonial culture.

### Metabolic changes upon spermatogonial differentiation

**Fig. 2:**
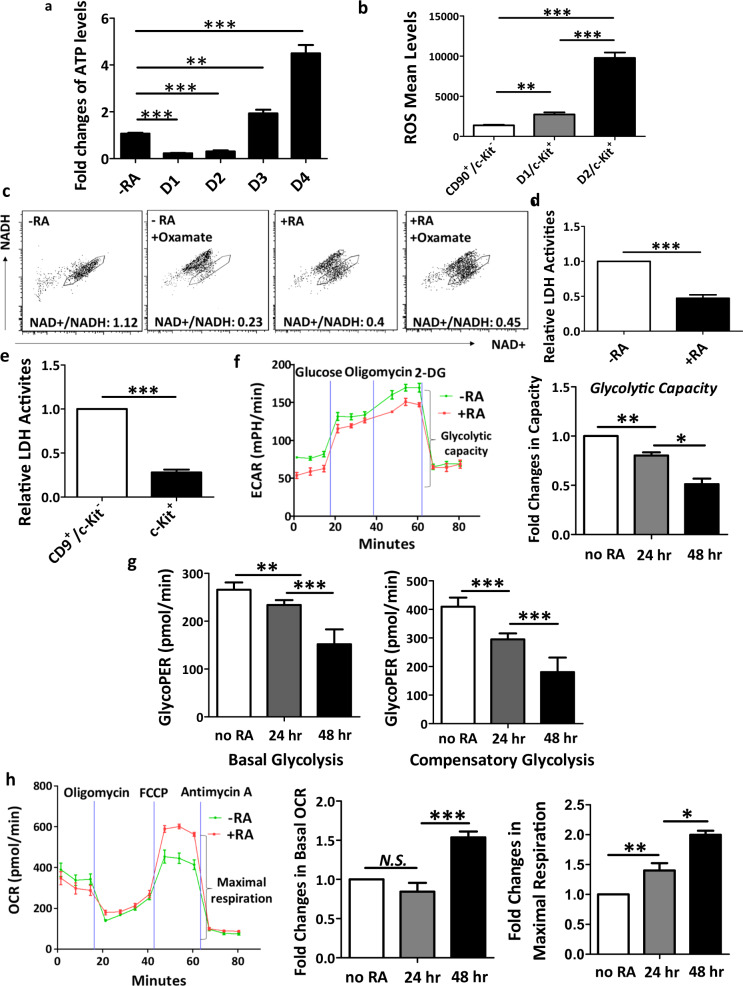
Distinct bioenergetic preference in undifferentiated and differentiating spermatogonia. **a** ATP levels were measured in undifferentiated spermatogonia (−RA) and in those treated with RA (+RA) for various time periods. **b** ROS levels were examined by flow cytometry in undifferentiated spermatogonia and c-Kit^+^ cells induced by RA treatment for one or two days. **c** Cytosolic NADH and NAD+ levels were examined by flow cytometry, and their ratios were calculated for in vitro cultured SoNar spermatogonia with or without RA for 24 h and/or oxamate pretreatment for 5 mins. **d**, **e** LDH activities were determined on undifferentiated spermatogonia and differentiating spermatogonia induced by RA treatment (**d**) or isolated by flow cytometry from mice at postnatal day 11 (**e**). **f**–**h** ECAR (**f**), GlycoPER (**g**), and OCR (**h**) were measured in undifferentiated spermatogonia and in spermatogonia after RA treatment for 24 or 48 h. **a**, **d**–**f**, **h** Fold changes relative to undifferentiated spermatogonia were calculated. **a**, **b**, **d**–**h** Data are presented as mean ± SEM. *P < 0.05; **P < 0.01; ***P < 0.001; *n* = 3 for **a**, **b**; *n* = 4 for **d**, **e**; *n* ≥ 5 for **f**, **h**.

To determine metabolic dynamics during spermatogonial differentiation, we first assessed ATP levels and ROS production along RA induction. We found that ATP levels were first decreased but upregulated significantly at day 3 post RA treatment (Fig. 2a), indicating a dynamical change in the balance of energy production and consumption during spermatogonial differentiation. Intriguingly, ROS levels started to increase at 24 h and were significantly elevated at 48 h post RA induction (Fig. 2b, Supplementary Fig. S1b), suggesting increased mitochondrial OXPHOS upon spermatogonial differentiation.

To further determine the changing metabolic preference during spermatogonial differentiation, we established a primary spermatogonial line containing a fluorescent cytosolic SoNar sensor, the intensity of which reflects the cytosolic NAD+ and NADH redox state^41^. In general, glycolysis produces NADH, which may be shuttled to mitochondria and oxidized to NAD+, or be recycled to NAD+ by lactate dehydrogenase (LDH) when generating lactate from pyruvate in the cytosol^42^. Therefore, the cytosolic NAD+/NADH ratio is determined by the net balance of these three pathways^42^. We found that undifferentiated spermatogonia displayed a much higher NAD+/NADH ratio than that of differentiating spermatogonia induced by RA for 24 h (Fig. 2c). Upon treatment of oxamate that blocks LDH activities, the NAD+/NADH ratio was dramatically reduced in undifferentiated spermatogonia (Fig. 2c), suggesting active conversions from pyruvate to lactate in those cells. By contrast, differentiating spermatogonia did not respond well to the oxamate inhibition (Fig. 2c). Because germ cells at distinct developmental stages may express different LDH isoforms^43,44^, we analyzed LDH activities in response to the oxamate inhibition and confirmed that in both groups LDH activities were efficiently inhibited by the oxamate treatment (Supplementary Fig. S1c). Consistent with these findings, when measured directly, we found significantly higher LDH activities in undifferentiated spermatogonia than in differentiating population treated with RA (Fig. 2d). We further confirmed these observations by assessing LDH activities on in vivo developed CD9^+^/c-Kit^−^ undifferentiated spermatogonia and c-Kit^+^ differentiating spermatogonia from wild-type mice (Fig. 2e, Supplementary Fig. S1d). In addition, we recently reported that ROS levels were significantly higher in c-Kit^+^ differentiating spermatogonia than those from CD90^+^/c-Kit^−^ undifferentiated spermatogonia developed in vivo^45^. These data thus support our findings that glycolysis decreases, but OXPHOS elevates upon spermatogonial differentiation under physiological conditions. Although interstitial somatic cells, such as Leydig cells from testes also express c-Kit^46^, these cells are largely stripped from seminiferous tubules with sequential trypsin and collagenase treatment during multistep spermatogonial isolation. Sorted c-Kit^+^ cells highly expressed germ cell markers, as displayed by real-time RT-PCR analyses (Supplementary Fig. S1e). Finally, to exclude the possibility that these altered metabolic changes were due to direct effects of RA treatment, we collected c-Kit^−^ and c-Kit^+^ cells from the same RA-treated population, and measured their LDH activities. Significantly higher LDH activities were detected in c-Kit^−^ spermatogonia than those from c-Kit^+^ cells (Supplementary Fig. S1f), thereby confirming that the observed metabolic shift is not an artifact from RA treatment, but rather determined by the developmental stages of spermatogonia. Taken together, these data reveal that the redox state of spermatogonia changes upon differentiation, and aerobic glycolysis is highly active in undifferentiated spermatogonia to enable the regeneration of NAD+ from pyruvate to lactate.

Using a Seahorse extracellular flux analyzer, we next examined the extracellular acidification rate (ECAR) in undifferentiated spermatogonia and differentiating populations after RA induction for 24 and 48 h. In principle, ECAR reflects both glycolysis and proton contribution from Krebs cycle^35^. We found that the glycolytic capacity was reduced in differentiating spermatogonia at 24 h and further decreased at 48 h post RA-induced differentiation, as calculated by the maximum change in ECAR after sequential addition of glucose, oligomycin (to inhibit OXPHOS), and 2-deoxyglucose (2-DG, to inhibit glycolysis; Fig. 2f, Supplementary Fig. S2a), suggesting a more active glycolysis in undifferentiated spermatogonia. To further distinguish the acidification effects of glycolysis from those generated through Krebs cycle, we measured proton efflux rate (PER) from undifferentiated spermatogonia and cells after RA induction in the presence of glucose, L-glutamine, and pyruvate with the sequential addition of rotenone/antimycin A (to block OXPHOS) and 2-DG. Both basal glycolysis (Fig. 2g, left panel) excluding the mitochondrion-derived acidification and compensatory glycolysis (Fig. 2g, right panel) were dropped gradually along differentiation, thereby supporting a higher glycolytic level in undifferentiated spermatogonia. In addition, we analyzed oxygen consumption rate (OCR) that represents the level of mitochondrial respiration. We found that basal OCR elevated dramatically in differentiating spermatogonia with RA treatment for 48 h (Fig. 2h, middle panel; Supplementary Fig. S2b). The maximum mitochondrial respiration, which was measured as the changes of OCR after sequential addition of oligomycin and FCCP followed by antimycin A supplementation, was significantly increased in cells at 24 and 48 h post RA treatment (Fig. 2h, right panel; Supplementary Fig. S2b), suggesting upregulated mitochondrial respiration upon spermatogonial differentiation. In summary, our data unveil a metabolic shift from glycolysis to mitochondrial respiration upon spermatogonial differentiation.

### Inhibition of glucose-6-phosphate production and pentose pathway in glycolysis represses spermatogonial differentiation

**Fig. 3:**
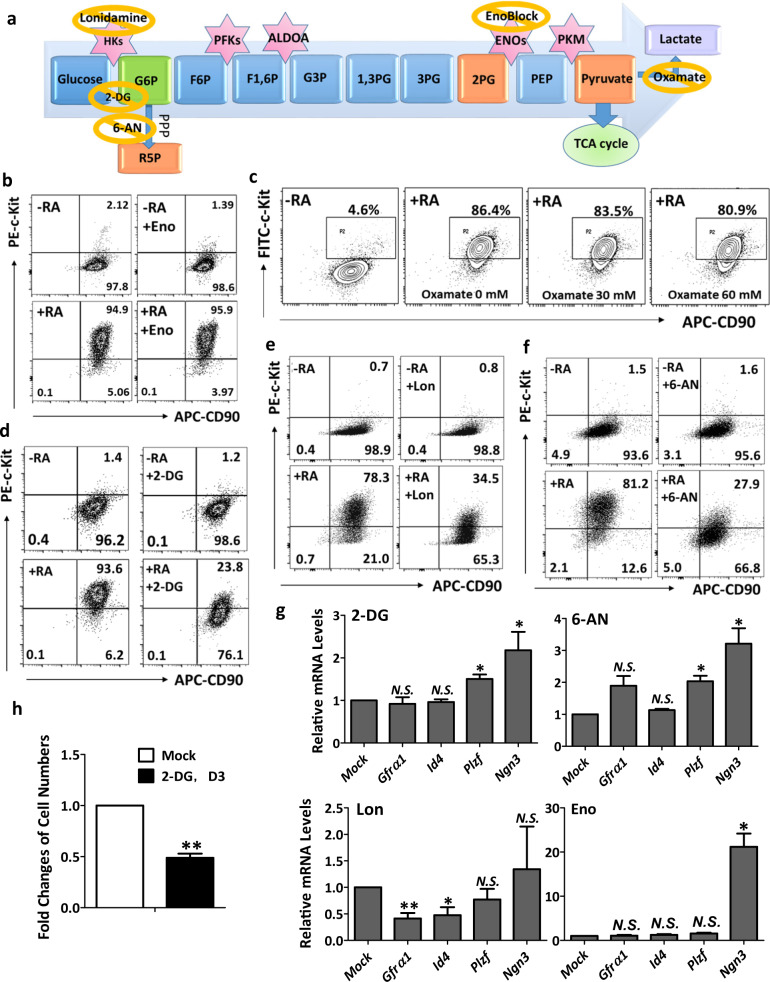
G6P formation from glucose in glycolysis is required for spermatogonial differentiation. **a** Overview of inhibitors used to block various steps of glycolysis. **b**–**f** The percentages of CD90^+^ and c-Kit^+^ cells were determined using flow cytometry on spermatogonia in the absence (−RA) or presence (+RA) of RA for 24 h. Inhibitors include enoblock (Eno; **b**), oxamate (**c**), 2-DG (**d**), loniamine (Lon; **e**), and 6-aminonicotinamide (6-AN; **f**). **g** Gene expression levels were measured by real-time RT-PCR on spermatogonia treated with inhibitors for 24 h. **h** The numbers of spermatogonia were counted in the presence of 10 mM 2-DG for three days (D3), and fold changes of cell numbers after 2-DG treatment was calculated in comparison to mock controls. **g**, **h** Data are presented as mean ± SEM. *P < 0.05; **P < 0.01; N.S., no statistical significance; *n* = 3.

To identify critical metabolic pathways for spermatogonial differentiation, we treated cells with inhibitors at various steps of glycolysis (Fig. 3a). Consistent with the observation above that differentiating spermatogonia prefer OXPHOS for energy production, enoblock, an inhibitor that blocks the formation from 2-phosphoglycerate to phosphoenol pyruvate had little effects on spermatogonial differentiation, as demonstrated by the similar percentage of c-Kit^+^ cells upon RA induction compared to mock controls (Fig. 3a, b). Similarly, oxamate that inhibited pyruvate to lactate conversion did not have any obvious influence on spermatogonial differentiation at a concentration of 60 mM or lower (Fig. 3a, c). At a high concentration of 120 mM, oxamate caused significant cell death likely due to chemical toxicity (data not shown).

Surprisingly, 2-DG, a glucose analog that inhibits the initial step of glycolysis from glucose to glucose-6-phosphate (G6P), significantly blocked spermatogonial differentiation within 24 h of treatment (Fig. 3a, d). The percentage of c-Kit^+^ cells upon RA induction dropped to 23.8% in 2-DG-treated cells, compared to 93.6% in the control group (Fig. 3a, d). Treatment of lonidamine, another inhibitor of hexokinase that catalyzes glucose to G6P, led to similar results (Fig. 3a, e), demonstrating that the formation of G6P is critical for spermatogonial differentiation. Because G6P provides building blocks for nucleotide synthesis through pentose phosphate pathway (PPP), we hypothesized that the reduced spermatogonial differentiation was likely due to a decreased supply of metabolites, such as ribose 5-phosphate for nucleotide synthesis. We tested this hypothesis with 6-aminonicotinamide, an inhibitor of G6P dehydrogenase that catalyzes G6P into 6-phosphogluconolactone at the first step of PPP (Fig. 3a). Indeed, 6-aminonicotinamide treatment significantly blocked spermatogonial differentiation, reducing c-Kit^+^ cells from 93.6% to 27.9% (Fig. 3f). Taken together, our data suggest that active G6P formation in glycolysis and PPP are required for spermatogonial differentiation.

Using these inhibitors at the same concentrations, we further examined their effects on spermatogonial maintenance. No obvious alteration in the percentage of CD90^+^/c-Kit^−^ undifferentiated spermatogonia was detected (Fig. 3b, d, f). We only observed a slightly reduced colony size after 24 h treatment of lonidamine (Supplementary Fig. S3a, b). As SSC population doubles about every five to six days^17^, short-term glycolysis inhibition may not lead to measurable defects in spermatogonial maintenance. Nevertheless, lonidamine treatment significantly decreased gene expression levels of *Gfrα1* and *Id4* (Fig. 3g), two SSC markers. Expression of *Ngn3*, a gene expressed mainly in early differentiating spermatogonia^13,47^, was elevated upon treatment of 2-DG, 6-aminonicotinamide, or enoblock (Fig. 3g). We further found that prolonged treatments of 2-DG for two and three days led to dramatic reductions in both spermatogonial colony size and cell number (Fig. 3h, Supplementary Fig. S4a), suggesting that inhibition of glycolysis also impairs spermatogonial self-renewal.

### Inhibition of OXPHOS blocks spermatogonial differentiation

**Fig. 4:**
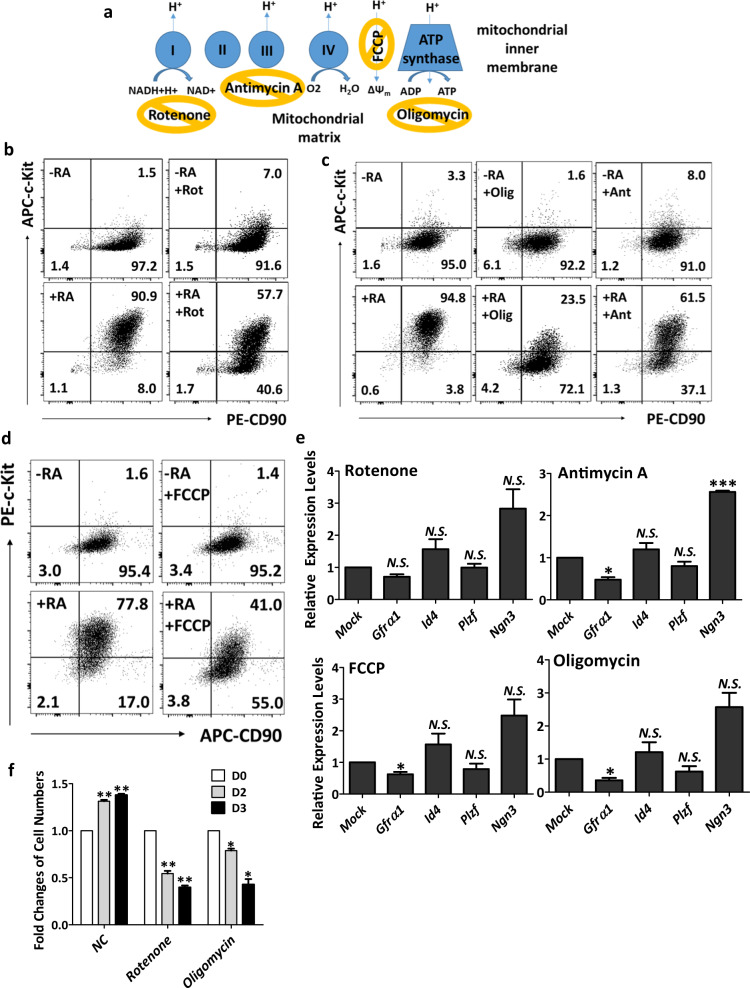
Spermatogonial differentiation needs OXPHOS. **a** Overview of inhibitors used to block various steps of mitochondrial respiration. **b**–**d** The percentages of CD90^+^ and c-Kit^+^ cells were analyzed by flow cytometry on in vitro cultured spermatogonia in the absence (−RA) or presence (+RA) of RA, with or without the following inhibitors: rotenone (Rot; **b**), antimycin A (Ant) and oligomycin (Olig; **c**), and FCCP (**d**). **e** Gene expression levels were measured by real-time RT-PCR assays on spermatogonia treated with inhibitors for 24 h. **f** The numbers of spermatogonia were counted in the presence of 6 μM rotenone or 2 μg/mL oligomycin for two and three days, and fold changes of cell numbers after the treatment of inhibitors were calculated in comparison to mock controls. **e**, **f** Data are presented as mean ± SEM. *P < 0.05; ***P < 0.001; N.S., no statistical significance; *n* = 3.

Using a similar strategy, we determined the requirement of specific enzyme complexes in OXPHOS during spermatogonial differentiation by targeting the mitochondrial respiration chain with various inhibitors. These inhibitors included rotenone against complex I, antimycin A inhibiting complex III, oligomycin repressing the function of ATP synthase, and FCCP, a chemical that disrupts ATP synthesis by abolishing the mitochondrial membrane potential (Fig. 4a). All these inhibitors significantly blocked the formation of c-Kit^+^ cells within 24 h post RA induction (Fig. 4b–d), supporting a critical requirement of OXPHOS in spermatogonial differentiation.

The same inhibitor treatments did not change the percentage of CD90^+^/c-Kit^−^ undifferentiated spermatogonia (Fig. 4b–d), and the spermatogonial morphology was normal after 24 h treatment (Supplementary Fig. S3b, c). We further examined the expression of genes that mark SSC identity by real-time RT-PCR. We found that rotenone treatment showed no effects, but antimycin A, FCCP, and oligomycin all decreased the expression levels of *Gfrα1* (Fig. 4e). Furthermore, prolonged treatment of rotenone or oligomycin for two and three days significantly reduced spermatogonial cell number and colony size (Fig. 4f, Supplementary Fig. S4b), suggesting mitochondrial respiration is needed for long-term maintenance of spermatogonial proliferation.

### Differential expression of metabolic regulators during spermatogonial differentiation

**Fig. 5:**
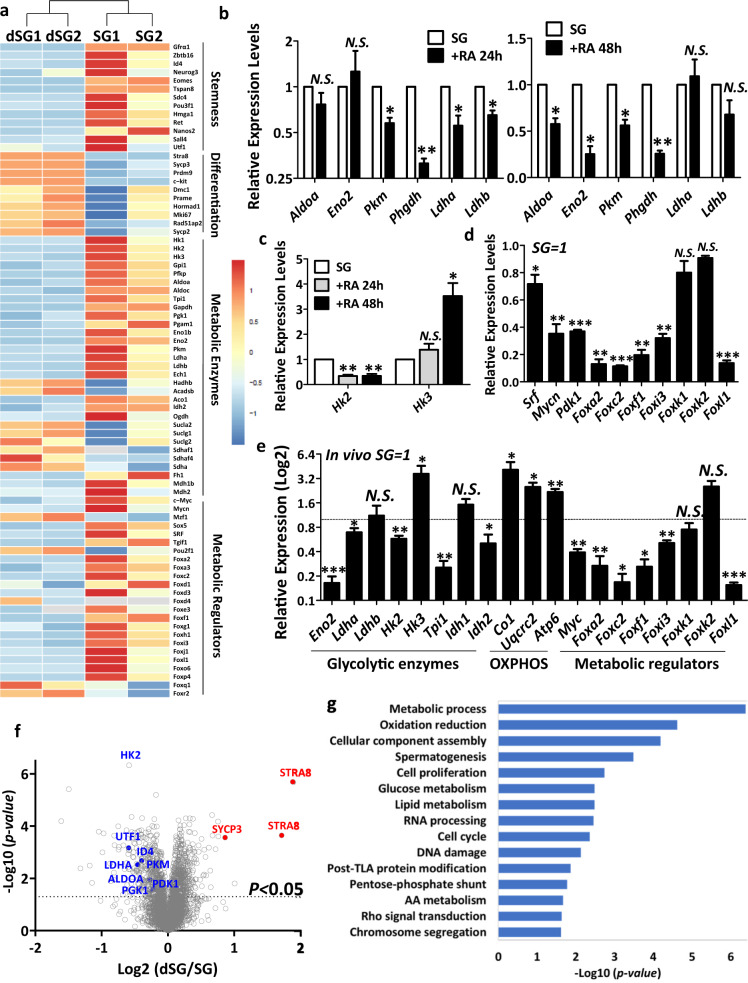
Differential expressions of metabolic regulators during spermatogonial differentiation. **a** A heat map graph extracted from RNA-seq analyses shows transcript levels of genes involved in spermatogonial stemness and differentiation, as well as those of metabolic enzymes, known metabolic regulators, and FOX family members. SG, undifferentiated spermatogonia without RA treatment; dSG, differentiating spermatogonia after RA treatment for 36 h. **b** Expression levels of enzymes that catalyze glycolysis were measured by real-time RT-PCR on spermatogonia in the absence or presence of RA for 24 h or 48 h. **c** Expression levels of hexokinase isoform 2 (*Hk2*) and 3 (*Hk3*) were measured by real-time RT-PCR on spermatogonia in the absence or presence of RA for 24 and 48 h. **d** Transcript levels of known metabolic regulators and selected FOX family members were examined by real-time RT-PCR. Their expression levels in differentiating spermatogonia induced by RA for 24 h were calculated relative to those in undifferentiated spermatogonia without RA treatment (SG). **e** Transcript levels of enzymes in glycolysis and mitochondrial OXPHOS, as well as metabolic regulators were assessed by real-time RT-PCR assays. Their expression levels in c-Kit^+^ cells from testes at postnatal day 12 were calculated relative to those in CD9^+^/c-Kit^−^ undifferentiated spermatogonia (SG) from the same mice. **f** Volcano plots generated from differentially expressed proteins between undifferentiated spermatogonia (SG) and spermatogonia with RA treatment for 36 h (dSG). Solid dots represent metabolic enzymes and markers genes for undifferentiated and differentiating spermatogonia with annotation. **g** Differentially expressed proteins are classified by GO analyses with DAVID online software. **b**–**e** Gene expression levels were normalized to β-actin expression of each sample and calculated relative to control groups. Data are presented as mean ± SEM from three or more independent experiments. *P < 0.05; **P < 0.01; ***P < 0.001; N.S., no statistical significance.

To systematically investigate the molecular mechanism underlying the metabolic changes during spermatogonial differentiation, we performed RNA-seq analyses to assess the genome-wide expression profiles of undifferentiated spermatogonia and the ones after 36-h RA induction. We confirmed that SSC markers, such as *Gfrα1, Id4, Plzf, Etv5*, and *Sall4*, were highly expressed in undifferentiated spermatogonia (Fig. 5a, Supplementary Table S1). By contrast, genes that indicate spermatogonial differentiation, such as *Sycp3, Stra8*, and *c-Kit*, were significantly increased upon RA induction (Fig. 5a, Supplementary Table S1). Interestingly, these two cell populations also showed dramatic differences in the levels of many metabolic enzymes, including those for glycolysis (e.g., HKs, Aldoa, *Eno1/2*, and *Ldha/b*; Fig. 5a, Supplementary Table S1). More importantly, several known metabolic regulators, such as *Pdk1/2*, *Srf*, *c-Myc*, and *Mycn*, were expressed at distinct levels in undifferentiated spermatogonia from RA-induced population (Fig. 5a, Supplementary Table S1). Consistent with these findings, gene ontology (GO) analyses using KEGG database revealed that glycolysis was among the top differentially regulated pathways between undifferentiated and differentiating spermatogonia (Supplementary Fig. S5a).

We further confirmed these RNA-seq results by real-time RT-PCR using total RNAs collected from independent experiments. The levels of several enzymes in glycolysis were indeed downregulated at different stages of spermatogonial differentiation (Fig. 5b). For example, expression levels of *Ldha* and *Ldhb*, enzymes that catalyze pyruvate to lactate conversion, were indeed decreased after 24-h RA induction (Fig. 5b), consistent with our observation that aerobic glycolysis for energy production is highly active in undifferentiated spermatogonia. Although *Ldha* and *Ldhb* transcript levels in differentiating spermatogonia at 48 h with RA treatment became comparable to undifferentiated spermatogonia (Fig. 5b), their protein levels or activities seemed to remain at lower levels as indicated by LDH activity measurement assays and ECAR from Seahorse analyses (Fig. 2), suggesting posttranscriptional regulation of LDH enzymatic activities. Notably, differential expression of hexokinase isoforms was also observed. The isoform *Hk2* was downregulated during the entire differentiation process (Fig. 5c); *Hk3* expression on the other hand remained unchanged at 24 h but was significantly upregulated after 48 h RA induction (Fig. 5c), suggesting differential regulatory mechanisms of stage-specific isozymes during spermatogonial differentiation. Interestingly, many members of the FOX family exhibited distinct expression profiles in undifferentiated spermatogonia from those under RA-induced differentiation (Fig. 5a, d). To confirm whether these enzymes and regulators display similar expression patterns during in vivo spermatogonial differentiation, we performed real-time RT-PCR on sorted CD9^+^/c-Kit^−^ undifferentiated and c-Kit^+^ differentiating spermatogonia from mouse testes. Indeed, we found that glycolytic enzymes expressed at higher levels in CD9^+^/c-Kit^−^ undifferentiated spermatogonia, while mitochondrial regulators were upregulated in c-Kit^+^ cells (Fig. 5e). In addition, several metabolic regulators (such as *Fox* genes and *Mycn*) displayed similar expression patterns during in vivo spermatogonial development to those found in vitro (Fig. 5e).

Because metabolic regulators and enzymes can also be regulated through protein translation, posttranslational modification, or differential expression of enzyme isoforms, transcript levels may reflect some but not all changes of these genes between undifferentiated and differentiating spermatogonia. To understand the changes in gene expression at the protein level during spermatogonial differentiation, we further performed quantitative proteomics on undifferentiated spermatogonia and RA-induced differentiating population (Supplementary Fig. S5b, c). A total of 4879 proteins were quantified, and 502 proteins displayed differential expression between these two groups (Supplementary Fig. S5d and Table S2). Consistent with our observation from RNA-seq analyses, several key enzymes in glycolysis, including HK2, ALDOA, PGK1, PKM, and LDHA, were significantly decreased in differentiating spermatogonia (Fig. 5f). The expression of PDK1, a kinase that inhibits citrate cycle via phosphorylating pyruvate dehydrogenase^48^, was also downregulated (Fig. 5f). Notably, GO enrichment analyses demonstrated that proteins involved in the metabolic process ranked at the top among all differentially regulated cellular processes between undifferentiated and differentiating spermatogonia (Fig. 5g). Taken together, our data reveal differential expression patterns of metabolic regulators between undifferentiated and differentiating spermatogonia at both transcript and protein levels, and these likely drive the metabolic shift from glycolysis to mitochondrial respiration during spermatogonial differentiation.

## Discussion

Previous studies report that increased glycolysis and hypoxia favor the establishment and long-term maintenance of SSCs (refs. ^35,39^), but ROS is also required for SSC self-renewal^38^. To date, no study has directly compared bioenergetic preference between undifferentiated spermatogonia and their differentiating derivatives, nor is clear whether undifferentiated spermatogonia mainly rely on aerobic glycolysis for energy production. Here, we demonstrate that spermatogonial maintenance needs both glycolysis and mitochondrial respiration. Inhibition of glycolysis and OXPHOS in undifferentiated spermatogonia leads to reduced spermatogonial colony size and decreased expressions of SSC marker genes. Glycolysis apparently provides ATP as well as building blocks for other cellular products, whereas OXPHOS likely generates ROS as a signaling molecule for spermatogonial self-renewal^38,49^. Nevertheless, consistent with what we know about other types of somatic stem cells, undifferentiated spermatogonia display a higher glycolytic capacity than their differentiating derivatives that prefer OXPHOS for energy production. We observed that spermatogonial differentiation was dramatically blocked by various inhibitors of mitochondrial respiration. Our data thus suggest that the levels of mitochondrial respiration and ROS production need to be critically regulated for proper spermatogonial proliferation and differentiation. It is plausible that spermatogonia need increased energy supply from OXPHOS to go through a transient proliferation to become differentiating progenitors that will further prepare for meiosis to form spermatocytes.

Although spermatogonia switch their preference for bioenergy production upon RA induction, differentiating spermatogonia still need glycolysis to produce various metabolic intermediates to get ready for meiosis. This is unique to germ cells, as somatic cells generally reduce or even stop proliferation upon differentiation and do not go through meiosis before maturation. We observed that 2-DG and lonidamine, two inhibitors that repress glucose to G6P conversion, significantly blocked spermatogonial differentiation. An increased *Hk3* expression likely compensates *Hk2* reduction during spermatogonial differentiation to meet the need of G6P formation from glycolysis for meiotic preparation. On the other hand, undifferentiated spermatogonia with prolonged treatment of 2-DG showed a significantly reduced number of spermatogonial cells. Lonidamine inhibition further decreased *Id4* and *Gfrα1* expression levels. These data are consistent with a previous report that 2-DG repressed long-term SSC growth and maintenance^35^. Interestingly, 2-DG treatment in spermatogonia upregulated *Ngn3* expression. Undifferentiated spermatogonia become c-Kit^+^ cells by going through a stage of committed progenitors, for which NGN3 is defined as a marker^13,47^. It is plausible that 2-DG blocks spermatogonial differentiation at the NGN3^+^ cell stage. Taken together, our data reveal that the initial step of glycolysis is crucial for both spermatogonial proliferation and differentiation from NGN3^+^ committed spermatogonial progenitors to c-Kit^+^ cells.

Mitochondrial respiration increases upon differentiation induced by RA treatment for 24 or 48 h, as supported by upregulated ROS levels and OCR, indicating that OXPHOS is particularly important for spermatogonial differentiation. Indeed, inhibitors of OXPHOS significantly reduced the formation of c-Kit^+^ differentiated cells. By contrast, obvious alteration in undifferentiated spermatogonia was observed mainly after prolonged treatments for two or three days. Mitochondrial respiration generally generates higher amounts of both ATP molecules and ROS than glycolysis. However, the ATP level dropped first and then elevated on day 3 during differentiation. The discrepancy between a declined ATP level and elevated ROS production suggests a higher energy consumption than production at the early stage of RA-induced differentiation, during which elevated energy demand is required for undifferentiated spermatogonial progenitors to go through transient and rapid proliferation before meiosis.

How do spermatogonia switch their bioenergetic preference from glycolysis to OXPHOS upon RA-induced differentiation? Our RNA-seq and proteomic data provided further clues to answer this question. We found that the metabolic process ranked among the top differentially altered pathways based upon GO enrichment analyses. Expression levels of key enzymes (HK2, ALDOA, PKM, and LDHA) in glycolysis were downregulated during differentiation at both RNA and protein levels. According to our transcriptome profiling, several known metabolic regulators, including *c-Myc* and *Mycn*, two genes that are critical for maintaining glycolysis in undifferentiated spermatogonia^35^, were decreased upon differentiation. Many FOX family members were also differentially expressed between undifferentiated spermatogonia and RA-induced cells. The FOX family of transcription factors is an evolutionarily ancient gene family. More than 40 FOX family members in mammals have been identified and are classified into subfamilies from FOXA to FOXP (ref. ^50^). Of these, FOXO subfamily members have been implicated as metabolic sensors in stem cell regulation^51^. Recently, FOXK1 and K2 were also reported to regulate aerobic glycolysis in somatic cells^52^. We did not observe any significant change in the expressions of *Foxk1* and *Foxk2* upon spermatogonial differentiation. However, we found six novel *Fox* genes that exhibited more than threefold differences in their expressions between undifferentiated and differentiating spermatogonia (Fig. 5d, Supplementary Table S1). RNA-seq analyses from published studies also showed a similar trend of differential *Fox* expression between undifferentiated and differentiating spermatogonia^53,54^. Our data thus suggest a potential requirement of these novel FOX members, as potential metabolic regulators of the bioenergetic switch during spermatogonial differentiation.

Admittedly, because culture conditions lack of physiological hypoxia environment, oxygen gradient across the testicular lumen, and supporting cells from the niche, studies of in vitro spermatogonial proliferation and differentiation cannot completely recapitulate what may happen in vivo. Nevertheless, our conclusion about a metabolic shift from glycolysis to OXPHOS during spermatogonial differentiation is supported by several lines of in vivo evidence. First, multiple RNA-seq analyses unveil that regulators in glycolysis are decreased in differentiating spermatogonia isolated from testes, while mitochondrial regulators appear to be upregulated, compared to SSCs (refs. ^34,53,54^). We observed the same phenomena by real-time RT-PCR on sorted undifferentiated spermatogonia and their progenitors developed in vivo. Second, we found that LDH activities decreased (Fig. 2e), but ROS levels^45^ elevated in c-Kit^+^ differentiating spermatogonia developed in vivo compared to CD9^+^/c-Kit^−^ undifferentiated population. These data strongly support that a metabolic shift indeed occurs in vivo during spermatogonial differentiation. Further research is warranted to investigate how environmental and developmental stimuli are hardwired with metabolic regulators to fine-tune the differentiation and self-renewal programs of SSCs during spermatogenesis.

## Materials and methods

### Experimental animals and establishment of spermatogonial cell culture

Testes were collected around postnatal day 5 from DBA mice or F1 of DBA crossed with GFP mice (Stock #: 003516, Jackson Lab, Sacramento, CA, USA). Spermatogonial culture was established and cultured according to published protocols^6^. Briefly, seminiferous tubules were incubated at 37 °C in PBS containing 1 mg/mL type IV collagenase (Thermo Fisher Scientific, Waltham, MA, USA) for 15 min and then in PBS/0.05% trypsin (Thermo Fisher Scientific) for 5 min, with occasional agitation. After enzyme digestion, CD9^+^/c-Kit^−^ undifferentiated spermatogonia were sorted by an Aria II flow cytometer (BD Bioscience, San Jose, CA, USA) and then maintained in Stempro34 (Thermo Fisher Scientific) supplemented with 20 ng/ml β-FGF (233-FB, R&D Systems, Minneapolis, MN,USA), 10^3^ U/mL ESGRO (Thermo Fisher Scientific), and 20 ng/mL GDNF/EGF (10561-HNCH/10605-HNAE, both from Sino Biological, Beijing, China). The use of animals and experimental protocols in this study were approved by the Institutional Animal Care and Use Committee of East China Normal University (project no. M20190317) and Michigan State University (08/17-137-00).

### Seminiferous tubule transplantation

Approximately 1 × 10^4^ (~3 µL as total volume) in vitro cultured GFP^+^ spermatogonia with trypan blue were transplanted into the seminiferous tubules of *KIT*^*W/W-v*^ testes (Jackson Lab) at three to five weeks of age. Mice were sacrificed two months later. GFP fluorescence in transplanted testes was examined under an Olympus microscope IX71, followed by histological analysis and immunohistofluorescence of testicular sections.

### Immunofluorescence, immunohistofluorescence, and histology studies

Cells on Matrigel (BD Bioscience) coated cover slides were fixed in 4% paraformaldehyde (Sangon Biotech, Shanghai, China) at 4 °C overnight, and immunofluorescence (IF) assays were performed according to published protocols^55^. The following antibodies were used: PLZF (SC-28319, Santa Cruz Biotech, Dallas, TX, USA), STRA8 (ab49602, Abcam, Cambridge, MA, USA), SYCP3 (ab15093, Abcam), Alexa Fluor 488- or TRITC-conjugated anti-mouse and anti-rabbit secondary antibodies (115-545-146, 115-025-146; 111-545-144, and 111-025-144, Jackson ImmunoResearch, West Grove, PA, USA). Nuclei were stained with 0.5 µg/mL DAPI before being visualized using an Olympus microscope BX53. Images were processed with the Image-J software. For histology studies, testes were fixed in Bouin’s fixative at 4 °C overnight for staining with hematoxylin and eosin, as previously described^56^. For immunohistofluorescence (IHF), testes were fixed with 4% paraformaldehyde in PBS at 4 °C overnight and embedded in paraffin. Testis sections were stained with an ACR antibody (HPA048687, Atlas Antibodies, Sweden), followed by staining with an Alexa Fluor 488-conjugated anti-rabbit secondary antibody (Jackson ImmunoResearch) and Rhodamine-labeled Peanut Agglutinin/PNA (RL-1072, Vector Laboratories, USA). Images were collected using a fluorescent microscope (Leica, DM400BLED368424).

### Spermatogonial differentiation and usages of inhibitors

Spermatogonial cells were maintained in Stempro34 medium with cytokines. Undifferentiated spermatogonia were transferred into Matrigel-coated dishes and spermatogonial differentiation was induced with RA (R2625, Sigma, St. Louis, MO, USA) in the spermatogonial culture medium. All RA treatments were performed with 100 nM RA for 24 h unless specified differently. To test the effects of inhibitors on spermatogonial proliferation, inhibitors were added at 12 h after cell passage. To examine the effects of inhibitors during spermatogonial differentiation, inhibitors were added simultaneously with RA treatment unless otherwise specified. The final concentration of inhibitors used in this study was listed as following unless otherwise specified: lonidamine (400 μM, HY-B0486) and AP-III-α4/ENOblock (20 µM, HY-15858) from MedChemExpress, Monmouth Junction, NJ, USA; 6-aminonicotinamide (1 mM C4497, APExBIO, Houston, TX, USA); 2-DG (10 mM, D8375); oxamate (30 μM, O2751), rotenone (12 μM, R8875), antimycin A (10 μM, A8674), and FCCP (20 μM, C2920) are from Sigma-Aldrich (St. Louis, MO, USA); and oligomycin A (2 µg/mLS1478, Selleck, Houston, TX, USA).

### Flow cytometry

Cells were digested into single cells using 0.05% trypsin and incubated in DMEM containing 10% fetal bovine serum (Thermo Fisher Scientific), stained with fluorochrome-conjugated antibodies, and washed with PBS before being analyzed, or sorted by a BD Fortessa or an Aria II flow cytometer (BD Biosciences). To determine the NAD+/NADH ratio, spermatogonia containing a SoNar probe were excited by 488 and 405 nm lasers, and fluorescence signals were detected through 530/30 FITC and 530/30 Alexa Fluor 430 channels, using BD Fortessa or Aria II flow cytometers. To determine ROS levels, cells were stained with 5 µM H_2_DCFDA (D399, Thermo Fisher Scientific) in PBS containing 1% BSA at 37 °C for 40 min in the dark followed by washing with PBS twice before measurement, using flow cytometers. Antibodies were used with 2 µg/mL per 1–2 million cells as a final working concentration: APC-CD9 (17-0091-82, eBioscience, USA); APC-CD90.2 (105311), and FITC-c-Kit (105806) from Biolegend, USA; and PE-cy7-CD90.2 (561642), PE-c-Kit (553355), and APC-c-Kit (553356) from BD Bioscience, USA.

### ATP measurement

Cellular ATP levels were measured using an ATP Assay Kit (Beyotime Biotech, Shanghai, China) according to the manufacturer’s instructions. Briefly, supernatants (60 µL) from cells were mixed with 60 µL of ATP detection buffer, and luminance signals were measured using an Infinite™ M200 Microplate Reader (TECAN, Männedorf, Switzerland). Protein concentration for each sample was determined by Bradford protein assay, and ATP concentration (µM) per mg of protein was calculated. Three or more technical replicates were examined for each independent experiment.

### LDH activity assay

To measure LDH activity, a Lactate Dehydrogenase Assay Kit (C0017, Beyotime Biotech, Shanghai, China) was used according to the manufacturer’s instructions. Briefly, cells were treated with 150 μL LDH release reagent and 120 μL supernatants were collected into a 96-well plate. After incubation with 60 μL LDH reaction reagent, absorbance was measured at the wavelength of 490 nm with a SPECTROstar Nano Microplate Reader (BMG Labtech, Germany). LDH activities (mU/ml) were calculated using standard curves generated from an LDH standard (L8080, Solarbio, Shanghai, China). Protein concentration for each sample was determined by Bradford protein assay, and LDH activities per mg of protein were calculated. To measure the inhibitory effects of oxamate, LDH activities were determined after incubation of cell lysate with 60 mM oxamate for 15 min on ice. Three or more technical replicates were used for each independent experiment.

### Seahorse analyses

For OCR and ECAR analyses, 2–3 × 10^4^ cells were plated on Matrigel-coated Seahorse XF96 microplates (Agilent Technologies, California, CA, USA, 101085-004). OCR, ECAR, and PER were determined using a XF96 extracellular flux analyzer (Seahorse Bioscience, Billerica, MA, USA). For OCR detection, injection port A on the sensor cartridge was loaded with 150 μM oligomycin A, port B with 200 μM FCCP, and port C with 100 μM antimycin A. During sensor calibration, cells were incubated at 37 °C in a CO_2_-free incubator with 180 μL assay medium (XF Base Medium with 10 mM glucose, 3.75 mM sodium pyruvate, and 2 mM L-glutamine). Basal respiration rates and maximum respiration were calculated following the manufacture’s protocols. For ECAR test, injection port A was loaded with 100 mM glucose, port B with 150 μM oligomycin, and port C with 100 mM 2-DG. Cells were incubated at 37 °C in 180 μL assay medium (XF Base Medium plus 2 mM L-glutamine) before measurement. Glycolytic capacity was calculated according to the manufacturer’s instructions. For PER detection, injection port A on the sensor cartridge was loaded with 100 μM rotenone and 100 μM antimycin A, and port B with 100 mM 2-DG. Cells were incubated at 37 °C in 180 μL of assay medium (XF DMEM with 10 mM glucose, 3.75 mM sodium pyruvate, and 2 mM L-glutamine) for 1 h and fresh medium was changed before measurement. Basal glycolysis, percentage of PER from glycolysis, and compensatory glycolysis were generated automatically through Seahorse Analyzer software. OCR, ECAR, and PER data were normalized to protein concentrations as determined by Bradford protein assay for each sample.

### RNA sequencing and bioinformatics analyses

For RNA-seq analyses, undifferentiated and differentiating spermatogonia were collected in Trizol (Thermo Fisher Scientific), and total RNAs were extracted. The cDNA was synthesized using a High-Capacity cDNA Reverse Transcription Kit (Applied Biosystems, Foster City, CA, USA) according to manufacturer’s instructions. RNA-seq libraries were generated with a NEB Next Directional RNA Library Prep Kit for Illumina® (New England Biolabs, Ipswich, MA, USA). Resulting libraries were size-selected by agarose gel electrophoresis and subsequently sequenced using an Illumina HiSeq-X platform with a 2 × 150 bp modality. Paired-end RNA-seq reads with 150 bp in each end were aligned to the *Mus musculus* genome (GRCm38) using Subread Aligner^57^ with its default parameter settings, and reads were counted using Feature Counts v1.5.3 (ref. ^58^). DESeq2 (ref. ^59^) was used to identify differentially expressed genes with false discovery rate (FDR) < 0.05 and fold change ≥ 2. KEGG enrichment pathway analyses were performed using KOBAS (ref. ^60^).

### Quantitative proteomics and bioinformatics analyses

For proteomic analyses, one million undifferentiated and differentiating spermatogonia (induced by RA for 36 h) were lifted from feeder cells with gentle pipetting and suspended in 100 µL cell lysis buffer (2% SDS, 100 mM NH_4_HCO_3_, protease inhibitors, and phosphatase inhibitors in PBS). Biological triplicates from each treatment group were collected. Cell lysis was processed through ultrasonication (Branson Sonifier 250, VWR Scientific, Batavia, IL), denaturing, and centrifugation to collect the supernatant. A total of 10 µg of proteins from each sample were utilized for reduction (by DL-Dithiothreitol) and alkylation (by Iodoacetamide). Single-spot solid-phase sample preparation with magnetic beads (SP3) was used to remove salts and SDS following a published protocol^61^. Labeled peptides were combined and processed by zip-tip desalting and high-pH reverse-phase liquid chromatography (RPLC) fractionation on an Easy nano-LC 1200 (Thermo Fisher Scientific) with a capillary column (75 μm i.d. × 50 cm, C18, 2 μm, 100 Å). Fractions were collected every 2 min with 400 nL of eluates into the tube containing the acidic aqueous phase. A total of 30 fractions were collected and analyzed by low-pH nanoRPLC-MS/MS with the same LC system as fractionation. A total of 80% of peptides from each fraction were loaded onto the analytical column (75 μm i.d. × 50 cm, C18, 2 μm, 100 Å) and then separated through a 3-h linear gradient with a flow rate at 200 nL/min. A Q-Exactive HF mass spectrometer (Thermo Fisher Scientific) was used for the MS/MS analysis with ESI voltage at 2 kV and MS parameters as follows: the resolution for full MS was 60,000, AGC at 3E6, the maximum injection time at 50 ms, and scan range at 300–1800 *m*/*z*. A Top10 data-dependent acquisition method was applied with following parameters: quadrupole isolation window was set at 2 *m*/*z*; normalized collision energy at 28 and 30%; MS/MS scan resolution at 30,000, AGC at 1e5, the maximum injection time at 50 ms, dynamic exclusion window at 30 s, fixed first mass at 100, and MS/MS intensity threshold for MS/MS was set 5e4. The database search was processed by Maxquant (v 1.5.5.1)^62^ with Uniport database for *M. musculus* (UP000000589). All parameters were set as default. Reporter ion MS2 was selected with TMT6plex for quantification. Filter by PIF was checked with 0.75 as minimum reporter PIF. The FDR was evaluated through the target–decoy database search. Reporter ion intensity of the first TMT channel (channel 126) was used to normalize reporter ion intensities of the other channels for fold change calculation: each individual reporter ion intensity was divided by the corresponding reporter ion intensity of the channel 126, converting the reporter ion intensity to protein ratio. Protein ratios of each TMT channel were divided by the corresponding median to make sure the ratios of each channel center at 1. The Perseus software was employed to generate volcano plots and perform *t*-test analyses^6^. The differentially expressed proteins between the differentiated and undifferentiated cells were determined with FDR at 0.1 and s0 at 0.1 using the Perseus software. GO terms of the differentially expressed proteins were analyzed by DAVID software^63^.

### RT-PCR and real-time PCR

Total RNAs were extracted using Trizol and reverse-transcribed using a cDNA Synthesis Kit (TaKaRa Biotechnology Co., Ltd., Shiga, Japan). Real-time PCR assays were performed and normalized to β-actin expression as previously described^64^ on a Biorad thermal cycler (Biorad, Hercules, CA, USA) or a Stratagene Mx3000P (Stratagene, San Diego, CA, USA). Sequences of primers used in this study are provided in Supplementary Table S3.

### Statistical analysis

Data were presented as mean ± SEM. All experiments were performed independently at least three times unless specified otherwise. Unpaired Student’s *t*-test (comparison between two groups) or one-way ANOVA (multiple groups) were conducted using the Prism Graphic software with the exception of RNA-seq analyses and proteomics.

## Supplementary information

Supplementary Information

Supplementary Table S1

Supplementary Table S2

Supplementary Table S3

## Data Availability

RNA-seq and proteomic raw data have been deposited in the publicly accessible database GEO (GSE150583) and ProteomeXchange (PXD019136).

## Supplementary information

**Supplementary Information** accompanies the paper at (10.1038/s41421-020-0183-x).

## References

[1] Wylie C (1999). Germ cells. Cell.

[2] Ewen KA, Koopman P (2010). Mouse germ cell development: from specification to sex determination. Mol. Cell Endocrinol..

[3] Griswold MD, Oatley JM (2013). Concise review: defining characteristics of mammalian spermatogenic stem cells. Stem Cells.

[4] Mecklenburg JM, Hermann BP (2016). Mechanisms regulating spermatogonial differentiation. Results Probl. Cell Differ..

[5] Yang QE, Oatley JM (2014). Spermatogonial stem cell functions in physiological and pathological conditions. Curr. Top. Dev. Biol..

[6] Kanatsu-Shinohara M (2003). Long-term proliferation in culture and germline transmission of mouse male germline stem cells. Biol. Reprod..

[7] Kubota H, Avarbock MR, Brinster RL (2004). Culture conditions and single growth factors affect fate determination of mouse spermatogonial stem cells. Biol. Reprod..

[8] Kanatsu-Shinohara M, Toyokuni S, Shinohara T (2004). CD9 is a surface marker on mouse and rat male germline stem cells. Biol. Reprod..

[9] Kubota H, Avarbock MR, Brinster RL (2003). Spermatogonial stem cells share some, but not all, phenotypic and functional characteristics with other stem cells. Proc. Natl Acad. Sci. USA.

[10] Buageaw A (2005). GDNF family receptor alpha1 phenotype of spermatogonial stem cells in immature mouse testes. Biol. Reprod..

[11] Chan F (2014). Functional and molecular features of the Id4+ germline stem cell population in mouse testes. Genes Dev..

[12] Costoya JA (2004). Essential role of Plzf in maintenance of spermatogonial stem cells. Nat. Genet..

[13] Kaucher AV, Oatley MJ, Oatley JM (2012). NEUROG3 is a critical downstream effector for STAT3-regulated differentiation of mammalian stem and progenitor spermatogonia. Biol. Reprod..

[14] Oatley MJ, Kaucher AV, Racicot KE, Oatley JM (2011). Inhibitor of DNA binding 4 is expressed selectively by single spermatogonia in the male germline and regulates the self-renewal of spermatogonial stem cells in mice. Biol. Reprod..

[15] Ikami K (2015). Hierarchical differentiation competence in response to retinoic acid ensures stem cell maintenance during mouse spermatogenesis. Development.

[16] Masaki K (2018). FGF2 has distinct molecular functions from GDNF in the mouse germline niche. Stem Cell Rep..

[17] Kubota H, Avarbock MR, Brinster RL (2004). Growth factors essential for self-renewal and expansion of mouse spermatogonial stem cells. Proc. Natl Acad. Sci. USA.

[18] Meng X (2000). Regulation of cell fate decision of undifferentiated spermatogonia by GDNF. Science.

[19] Anderson EL (2008). Stra8 and its inducer, retinoic acid, regulate meiotic initiation in both spermatogenesis and oogenesis in mice. Proc. Natl Acad. Sci. USA.

[20] Bowles J (2006). Retinoid signaling determines germ cell fate in mice. Science.

[21] Oatley JM, Avarbock MR, Telaranta AI, Fearon DT, Brinster RL (2006). Identifying genes important for spermatogonial stem cell self-renewal and survival. Proc. Natl Acad. Sci. USA.

[22] Yang F (2006). Mouse SYCP2 is required for synaptonemal complex assembly and chromosomal synapsis during male meiosis. J. Cell Biol..

[23] Yuan L (2000). The murine SCP3 gene is required for synaptonemal complex assembly, chromosome synapsis, and male fertility. Mol. Cell..

[24] Brinster RL, Avarbock MR (1994). Germline transmission of donor haplotype following spermatogonial transplantation. Proc. Natl Acad. Sci. USA.

[25] Ogawa T, Dobrinski I, Avarbock MR, Brinster RL (2000). Transplantation of male germ line stem cells restores fertility in infertile mice. Nat. Med..

[26] Ohta H, Tohda A, Nishimune Y (2003). Proliferation and differentiation of spermatogonial stem cells in the w/wv mutant mouse testis. Biol. Reprod..

[27] Ryall JG, Cliff T, Dalton S, Sartorelli V (2015). Metabolic reprogramming of stem cell epigenetics. Cell. Stem Cell..

[28] Wu J, Ocampo A, Belmonte JCI (2016). Cellular metabolism and induced pluripotency. Cell.

[29] Folmes CD (2011). Somatic oxidative bioenergetics transitions into pluripotency-dependent glycolysis to facilitate nuclear reprogramming. Cell Metab..

[30] Zhang J, Nuebel E, Daley GQ, Koehler CM, Teitell MA (2012). Metabolic regulation in pluripotent stem cells during reprogramming and self-renewal. Cell. Stem Cell..

[31] Shyh-Chang N, Daley GQ, Cantley LC (2013). Stem cell metabolism in tissue development and aging. Development.

[32] Wang K (2013). Redox homeostasis: the linchpin in stem cell self-renewal and differentiation. Cell Death Dis..

[33] Green CD (2018). A comprehensive roadmap of murine spermatogenesis defined by single-cell RNA-seq. Dev. Cell..

[34] Hermann BP (2018). The mammalian spermatogenesis single-cell transcriptome, from spermatogonial stem cells to spermatids. Cell Rep..

[35] Kanatsu-Shinohara M (2016). Myc/Mycn-mediated glycolysis enhances mouse spermatogonial stem cell self-renewal. Genes Dev..

[36] Cross BA, Silver IA (1962). Neurovascular control of oxygen tension in the testis and epididymis. J. Reprod. Fertil..

[37] Wenger RH, Katschinski DM (2005). The hypoxic testis and post-meiotic expression of PAS domain proteins. Semin Cell Dev. Biol..

[38] Morimoto H (2013). ROS are required for mouse spermatogonial stem cell self-renewal. Cell. Stem Cell..

[39] Helsel AR, Oatley MJ, Oatley JM (2017). Glycolysis-optimized conditions enhance maintenance of regenerative integrity in mouse spermatogonial stem cells during long-term culture. Stem Cell Rep..

[40] Holley RJ (2015). Comparative quantification of the surfaceome of human multipotent mesenchymal progenitor cells. Stem Cell Rep..

[41] Zhao Y (2015). SoNar, a highly responsive NAD+/NADH Sensor, allows high-throughput metabolic screening of anti-tumor agents. Cell Metab..

[42] Hao X (2019). Metabolic imaging reveals a unique preference of symmetric cell division and homing of leukemia-initiating cells in an endosteal niche. Cell Metab..

[43] Boussouar F, Benahmed M (2004). Lactate and energy metabolism in male germ cells. Trends Endocrinol. Metab..

[44] Meistrich ML, Trostle PK, Frapart M, Erickson RP (1977). Biosynthesis and localization of lactate dehydrogenase X in pachytene spermatocytes and spermatids of mouse testes. Dev. Biol..

[45] Chen W (2020). MFN2 plays a distinct role from MFN1 in regulating spermatogonial differentiation. Stem Cell Rep..

[46] Rothschild G (2003). A role for kit receptor signaling in Leydig cell steroidogenesis. Biol. Reprod..

[47] Song HW, Wilkinson MF (2014). Transcriptional control of spermatogonial maintenance and differentiation. Semin Cell Dev. Biol..

[48] Kolobova E, Tuganova A, Boulatnikov I, Popov KM (2001). Regulation of pyruvate dehydrogenase activity through phosphorylation at multiple sites. Biochem J..

[49] Bigarella CL, Liang R, Ghaffari S (2014). Stem cells and the impact of ROS signaling. Development.

[50] Hannenhalli S, Kaestner KH (2009). The evolution of Fox genes and their role in development and disease. Nat. Rev. Genet..

[51] Liang R, Ghaffari S (2018). Stem cells seen through the FOXO lens: an evolving paradigm. Curr. Top. Dev. Biol..

[52] Sukonina V (2019). FOXK1 and FOXK2 regulate aerobic glycolysis. Nature.

[53] Garbuzov A (2018). Purification of GFRalpha1+ and GFRalpha1- spermatogonial stem cells reveals a niche-dependent mechanism for fate determination. Stem Cell Rep..

[54] Hammoud SS (2015). Transcription and imprinting dynamics in developing postnatal male germline stem cells. Genes Dev..

[55] Zhang J (2016). GASZ and mitofusin-mediated mitochondrial functions are crucial for spermatogenesis. EMBO Rep..

[56] Wang J (2017). NRF1 coordinates with DNA methylation to regulate spermatogenesis. FASEB J..

[57] Liao Y, Smyth GK, Shi W (2013). The Subread aligner: fast, accurate and scalable read mapping by seed-and-vote. Nucleic Acids Res..

[58] Liao Y, Smyth GK, Shi W (2014). featureCounts: an efficient general purpose program for assigning sequence reads to genomic features. Bioinformatics.

[59] Love MI, Huber W, Anders S (2014). Moderated estimation of fold change and dispersion for RNA-seq data with DESeq2. Genome Biol..

[60] Xie C (2011). KOBAS 2.0: a web server for annotation and identification of enriched pathways and diseases. Nucleic Acids Res..

[61] Hughes CS (2014). Ultrasensitive proteome analysis using paramagnetic bead technology. Mol. Syst. Biol..

[62] Cox J, Mann M (2008). MaxQuant enables high peptide identification rates, individualized p.p.b.-range mass accuracies and proteome-wide protein quantification. Nat. Biotechnol..

[63] Huang da W, Sherman BT, Lempicki RA (2009). Systematic and integrative analysis of large gene lists using DAVID bioinformatics resources. Nat. Protoc..

[64] Zhang X (2014). Transcriptional repression by theS BRG1-SWI/SNF complex affects the pluripotency of human embryonic stem cells. Stem Cell Rep..

