# Supplementary material for: A bioenergetic shift is required for spermatogonial differentiation
Source: Cell Discov. 2020 Aug 18;6:56. doi: 10.1038/s41421-020-0183-x (PMC7431567; doi:10.1038/s41421-020-0183-x)
Supplement: Supplementary file 1 — Supplementary Information [file 41421_2020_183_MOESM1_ESM.pdf]

Supplementary Figure Legend

Fig. S1. A distinct bioenergetic preference in undifferentiated and differentiating spermatogonia. (a) Expression levels of *Stra8* were measured by real-time RT-PCR in spermatogonia treated with various RA concentration. n-=3 **(b)** An example of histograms of ROS levels measured by flow cytometry in undifferentiated spermatogonia and spermatogonia treated with RA for 24 or 48 hrs. (**c**) LDH activities were determined in cell lysates from undifferentiated (-RA) and differentiating (+RA) spermatogonia pre-treated with oxamate and normalized to those without oxamate inhibition. (**d**) Colorimetric display of LDH activities determined on 1x10e5 $CD9^+$/c-Kit^- undifferentiated and c-Kit^+ differentiating spermatogonia from P11 mice. (**e**) Real-time RT-PCR on sorted c-Kit^+ cells from P12 testes. (**a, c, e**) Data are presented as mean ± SEM from four independent experiments. * $P < 0.05$; ** $P < 0.01$. Relative gene expression levels were normalized to β-actin expression and calculated in comparison to control group. (**f**) An example of flow cytometry on c-Kit^+ cells induction by different RA concentration. Right panel shows a colorimetric display of LDH activities, including the ones determined on c-Kit^- and c-Kit^+ cells from the same 0.1 nM RA-treated population.

Fig. S2. Undifferentiated spermatogonia have higher glycolysis than and differentiating spermatogonia induced by RA treatment, analyzed by Seahorse metabolic assays. (a-b) ECAR (**a**) and OCR (**b**) were measured in undifferentiated spermatogonia and in spermatogonia after RA treatment for 48 hrs. Data are presented as mean ± SEM. n≥5.

Fig. S3. Inhibition of bioenergetics impacts spermatogonial self-renewal and differentiation. (a-c) Morphology of spermatogonial colonies in the absence or presence of RA for 24-hr, with concomitant treatment of inhibitors for glycolysis (**a**), an LDH inhibitor Oxamate or Rotenone to repress OXPHOS complex I (**b**), or with inhibitors to repress various steps of OXPHOS (**c**). Scale

Bar: 200 μm. Insets are blow-up images with higher magnificent powers. (**a**) Enoblock: Eno; Lonidamine: Lon; 6-Aminonicotinamide: 6-AN. Working concentration of inhibitors was provided in the method.

Fig. S4. Prolonged inhibition of bioenergetics impacts spermatogonial proliferation. (**a-b**) Morphology of spermatogonial colonies in the absence or presence of RA and various inhibitors of glycolysis (**a**) or OXPHOS (**b**) after 2 to 3 days of treatments. Reduced doses of inhibitors were used for Rotenone (6 μM) and Oligomycin (2 μg/ml). Scale Bar: 200 μm. Insets are blow-up images with higher magnificent powers.

Fig. S5. Metabolic regulators are differentially expressed in undifferentiated and differentiating spermatogonia. (**a**) Pathway analyses of RNA-seq results using KEGG databases. (**b**) Flow cytometry analyses of CD9 and c-Kit staining on samples that were submitted for proteomic analyses. (**c**) Marker proteins detected by proteomics. N=3. * $P < 0.05$. (**d**) The numbers of proteins that are differentially expressed between undifferentiated and differentiating spermatogonia.

Supplementary Table S1. RNA-seq analyses of undifferentiated (SG) and differentiating (dSG) spermatogonia

Supplementary Table S2. Proteomics of undifferentiated (SG) and differentiating (dSG) spermatogonia

Supplementary Table S3. Primer sequences used in this study

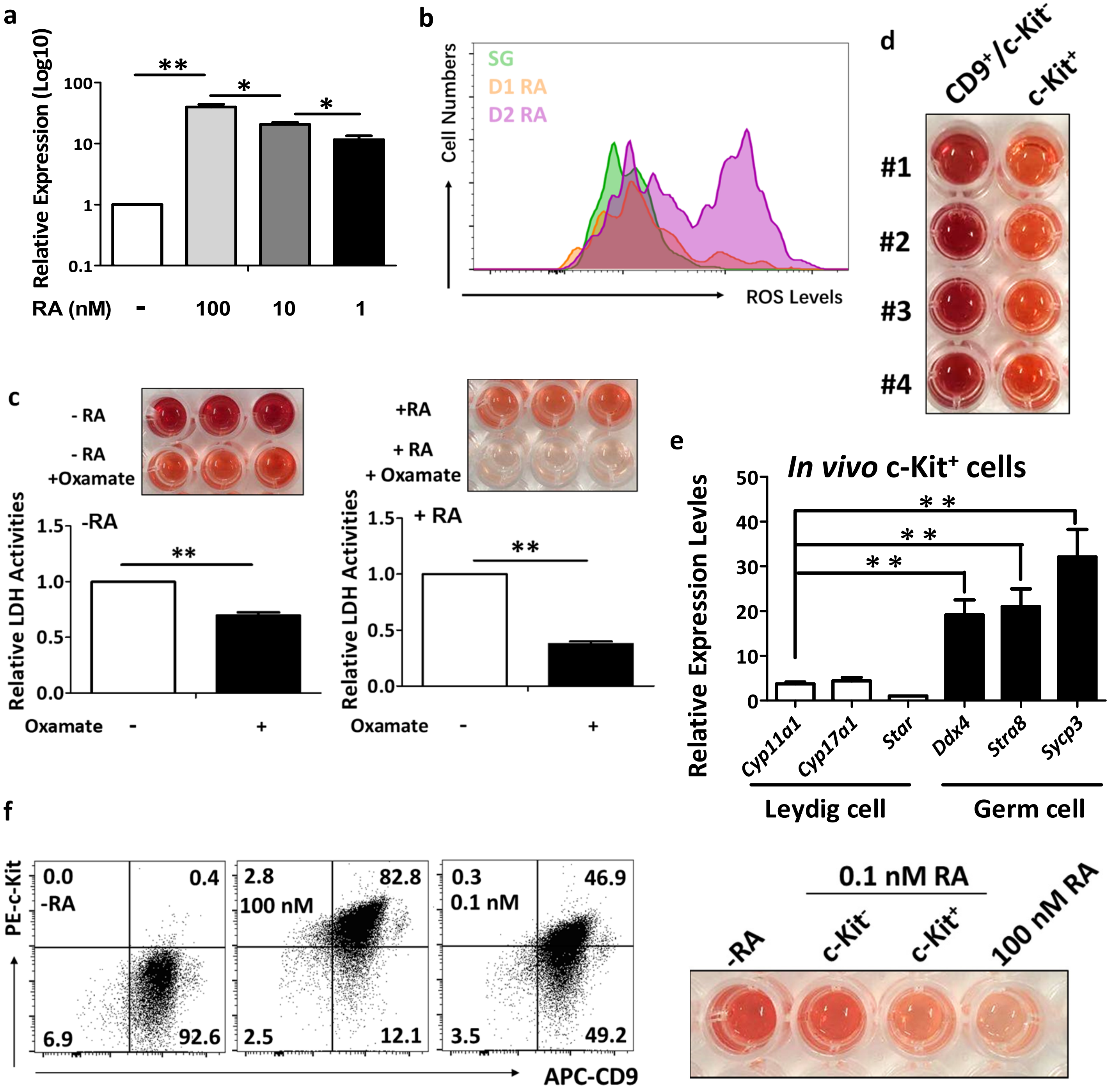

Supplementary Fig. S1

a

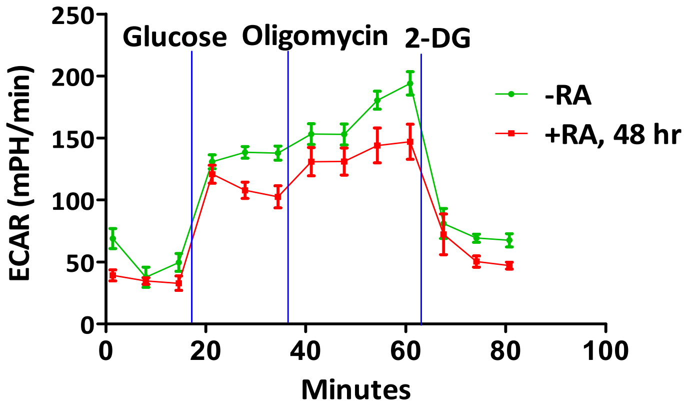

b

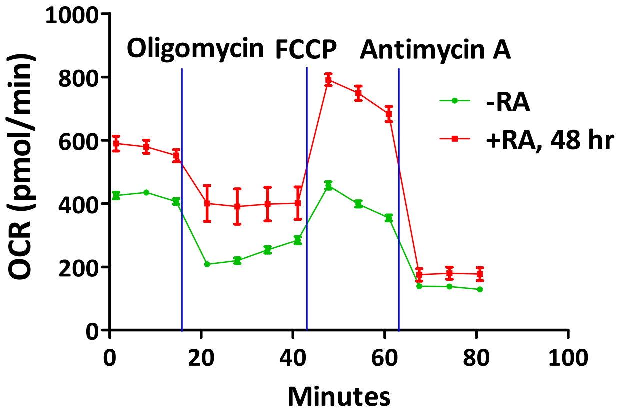

Supplementary Fig. S2

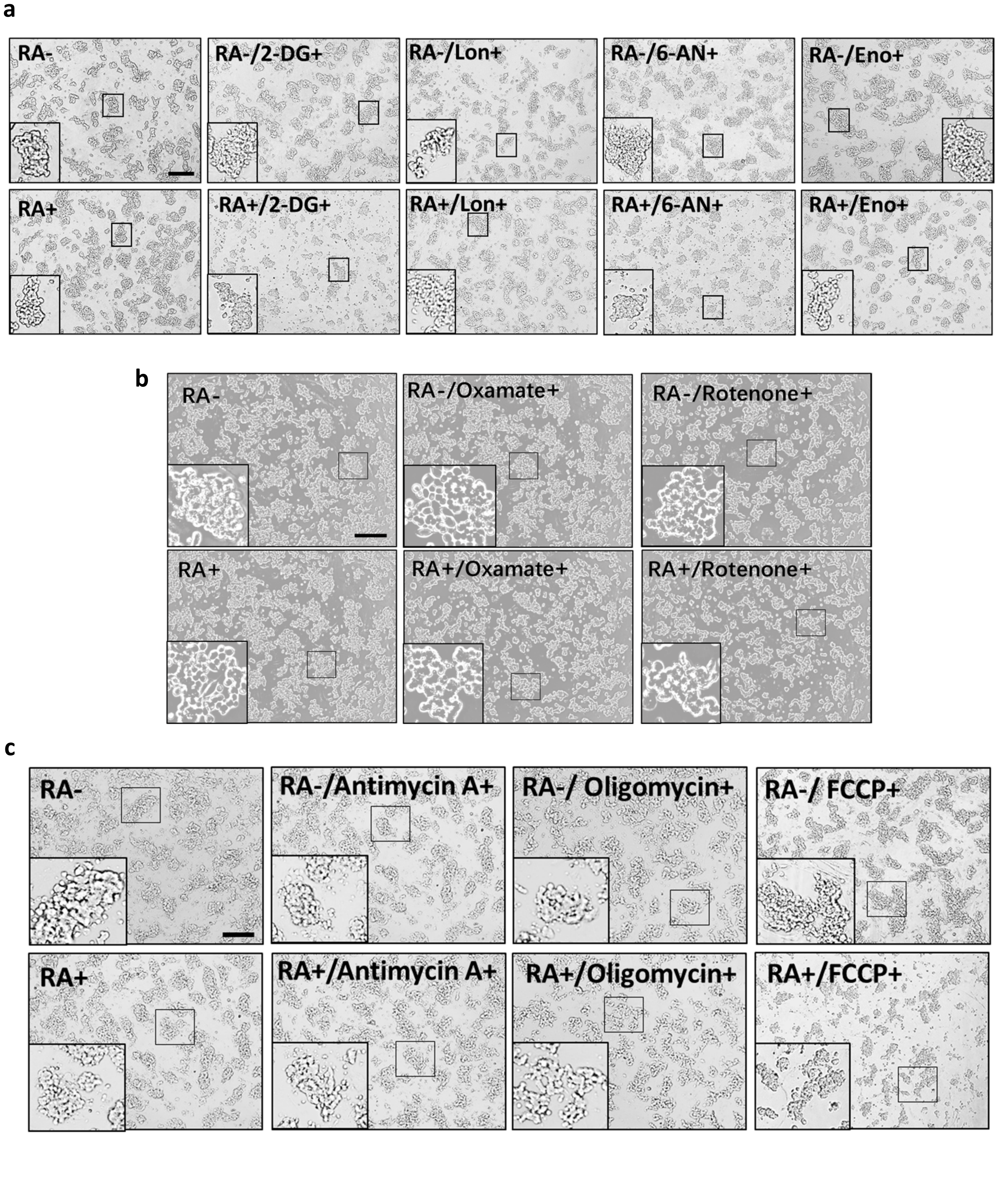

Supplementary Fig. S3

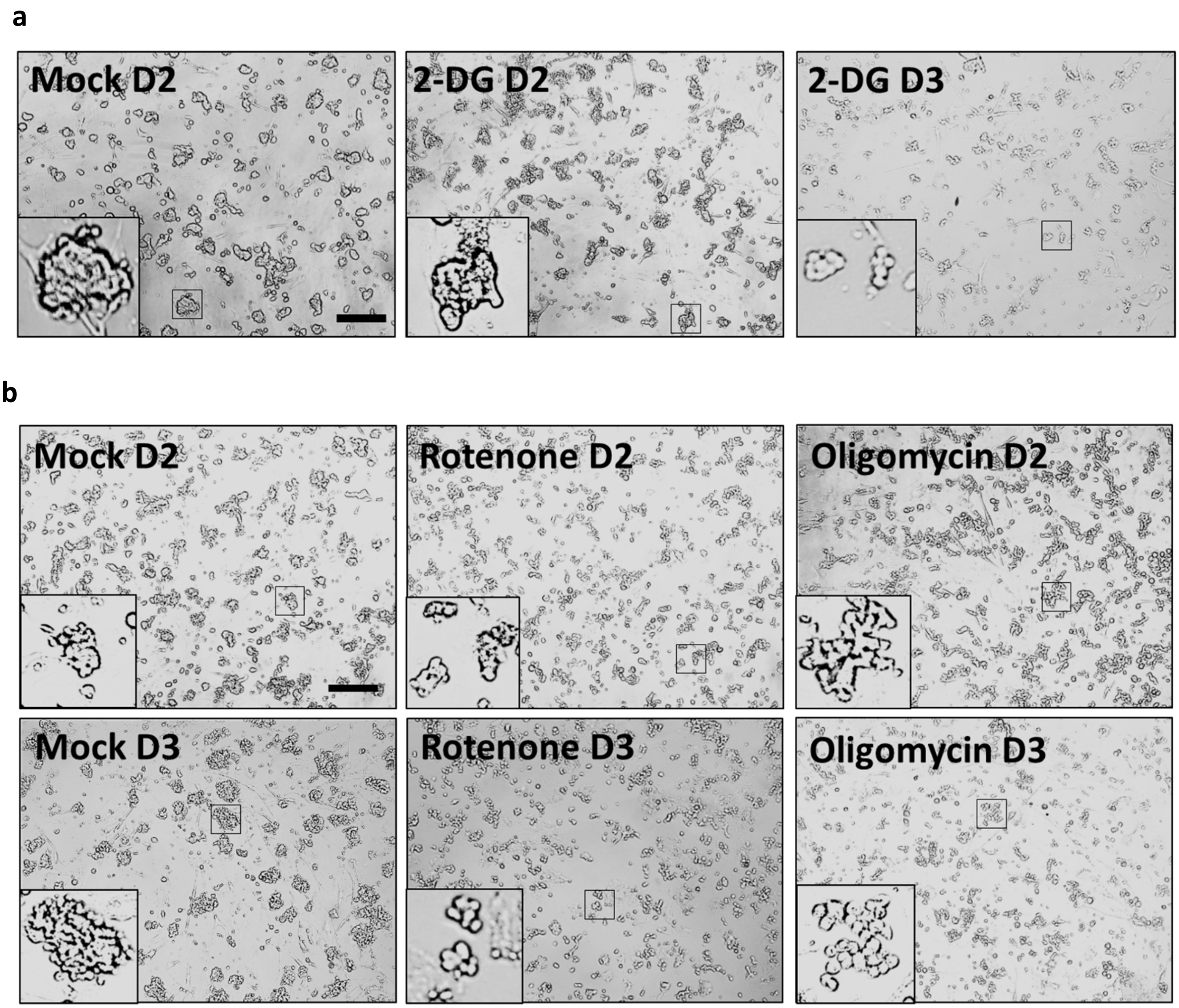

Supplementary Fig. S4

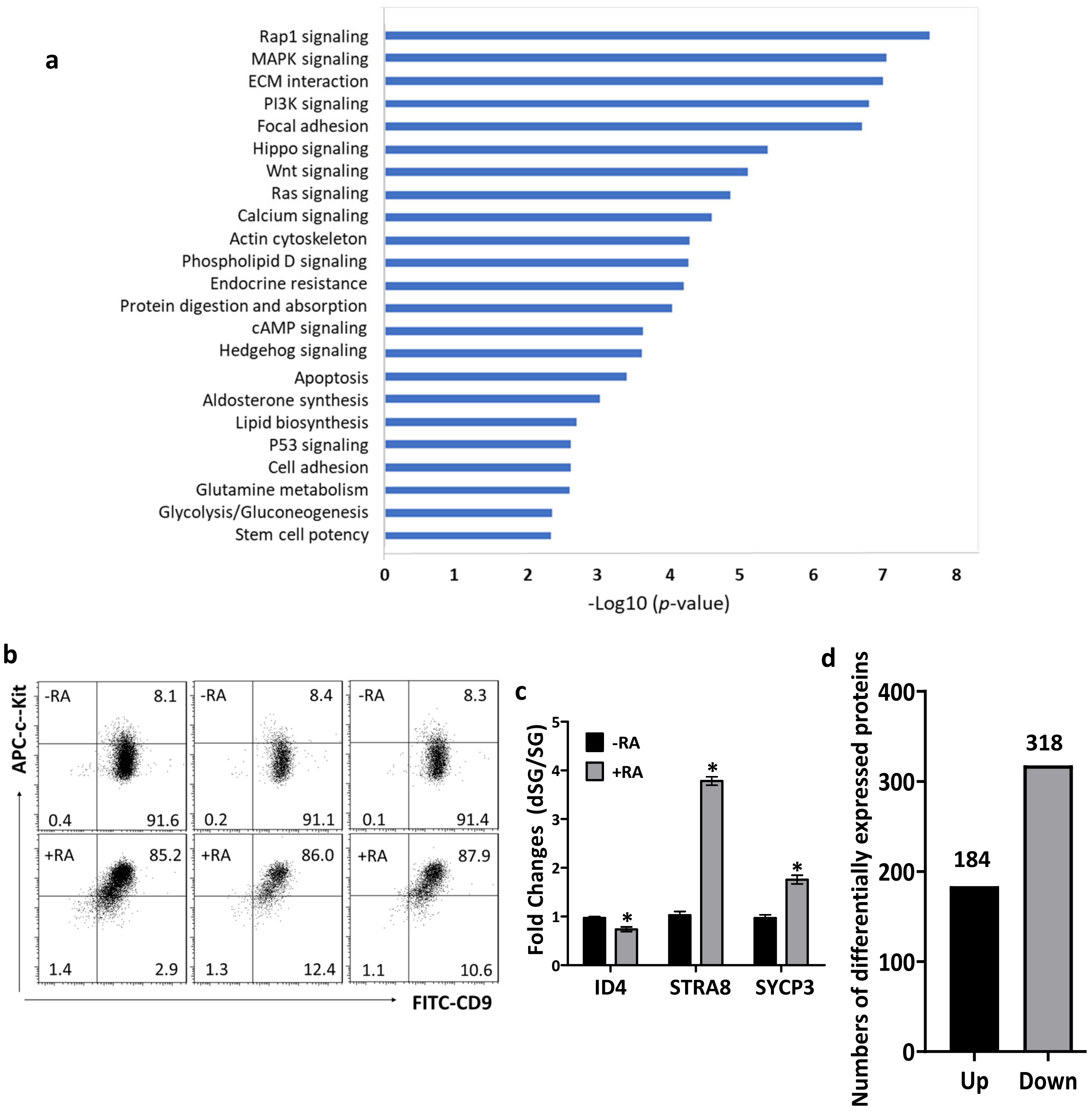

Supplementary Fig. S5
